# Nuclear disarmament verification via resonant phenomena

**DOI:** 10.1038/s41467-018-03680-4

**Published:** 2018-03-28

**Authors:** Jake J. Hecla, Areg Danagoulian

**Affiliations:** Department of Nuclear Science and Engineering, Massachusetts Institute of Technology, 77 Massachusetts Avenue, Cambridge, MA 02139 USA

## Abstract

Nuclear disarmament treaties are not sufficient in and of themselves to neutralize the existential threat of the nuclear weapons. Technologies are necessary for verifying the authenticity of the nuclear warheads undergoing dismantlement before counting them toward a treaty partner’s obligation. Here we present a concept that leverages isotope-specific nuclear resonance phenomena to authenticate a warhead’s fissile components by comparing them to a previously authenticated template. All information is encrypted in the physical domain in a manner that amounts to a physical zero-knowledge proof system. Using Monte Carlo simulations, the system is shown to reveal no isotopic or geometric information about the weapon, while readily detecting hoaxing attempts. This nuclear technique can dramatically increase the reach and trustworthiness of future nuclear disarmament treaties.

## Abstract (web summary)

Authenticating a nuclear warhead without revealing its design is a challenge. Here the authors discuss a nuclear disarmament verification method based on neutron resonance analysis which is sensitive to the isotopic composition of the materials used in warheads.

## Introduction

The abundance of nuclear weapons could be one of the biggest threats to global security and stability. Currently Russia and the United States own more than 90% of all nuclear weapons. It is estimated that as many as 14 000 units are part of their arsenals of retired and stockpiled weapons, and an additional 3700 units make up the deployed arsenals^1,2^. Such large numbers expose the world to the danger of catastrophic devastation in case of an intentional or accidental nuclear war, and also raise the danger of nuclear terrorism and nuclear proliferation due to possible theft or loss of nuclear weapons. So far most treaties, such as the Strategic Arms Reduction Treaty (New START) and the Intermediate-Range Nuclear Forces treaty, stipulate cross-verification of the dismantlement of delivery systems—such as bomber aircraft and cruise missiles. The delivery methods are a reliable proxy of strike capability in a nuclear war scenario. However, a reduction effort that is limited only to the verification of delivery methods leaves behind the problem of large stockpiles of surplus nuclear warheads. Thus, disarmament treaties that target the stockpiles themselves are indispensable for reducing this combined danger. To enable such treaties, however, new technologies are necessary to achieve treaty verification while protecting the secrets of the treaty participants. This means verifying the authenticity of nuclear weapons—before their destruction is counted toward a treaty participant’s obligations—without revealing any classified information. Such a technique will be a highly powerful tool in enacting far-reaching disarmament treaties.

But how does one verify that an object is a weapon without inspecting its interior? This apparent paradox has puzzled policy makers and researchers alike for the last few decades, with no clear solutions adopted. Past United States–Russia lab-to-lab collaboration included the research and development of information barriers^3^. These are devices that rely on software and electronics to analyze data from radiation detectors and compare the resulting signal against a set of attributes in a so-called attribute verification scheme^4^. The attributes can be the plutonium mass and enrichment, the presence of explosives, etc. Most importantly, these attributes have to be quite broad, to prevent the release of classified information. This in its turn makes them insensitive to a variety of hoaxing scenarios. The other major difficulty of this paradigm is that it shifts the problem of verification to software and electronics, whose components themselves will need to undergo verification and validation for the possible presence of spyware, back-door exploits, and other hidden functionality. If present, these can either leak secret information to the inspectors, or clear fake warheads. To overcome this, an alternative approach called template verification has been proposed where all candidate warheads and/or their components are compared to those from a previously authenticated template. The authenticated template itself can be selected based on situational context: a random warhead acquired from a deployed intercontinental balistic missile (ICBM) during a surprise visit by the inspection crew can be expected to be real, since a country’s nuclear deterrence hinges on having real warheads on their ICBM. To strengthen the confidence in its authenticity, multiple warheads can be removed from multiple ICBM and later compared to each other using the verification protocol described in this work. For a history of early attribute and template verification research see Yan and Glaser^5^.

While significant first steps were taken in physical cryptographic template verification research, much needs to be done in insuring that the verification protocol is both hoax-resistant as well as information-secure. A template verification method based on the non-resonant transmission of fast neutrons was developed by researchers at Princeton^6,7^. Independently, an isotope-sensitive physical cryptography system was proposed by researchers, including one of the authors of this paper, at Massachusetts Institute of Technology’s (MIT’s) Laboratory of Nuclear Security and Policy^8–10^. Both approaches have advantages and disadvantages. The Princeton concept has strong information security in the form of a zero-knowledge (ZK) proof. It relies primarily on the non-resonant scattering of fast neutrons, a process that is almost identical for most actinides, making it prone to some isotopic hoaxes. An example of such a hoaxing scenario is the replacement of the weapon-grade plutonium (WGPu) pit, which in this work refers to the hollow plutonium sphere at the center of a fission nuclear weapon, with more easily available reactor-grade plutonium (RGPu). Current work is being performed to mitigate this vulnerability by using the detection of fission neutrons to differentiate between fissile and fissionable materials^11^. The previous MIT system, relying on isotope-specific nuclear resonance fluorescence (NRF) signatures offers strong hoax resistance. However, it is not fully ZK, and thus needs to undergo thorough checks for information security. The methodology described in this work combines the strengths of the Princeton concept (ZK proof) with the strengths of the MIT concept (isotopic sensitivity), while avoiding their weaknesses. Such an outcome can have a strong impact on future arms reduction treaties.

This technique uses resonance phenomena to achieve isotope-specific data signatures, which can be used to obtain a unique fingerprint of the object. This is achieved by exploiting nuclear resonances in actinides when interacting with epithermal neutrons in the 1–10 eV range. Unlike fast neutrons, which do not have this isotope-specificity for high Z nuclei, the epithermal neutron transmission signal can be made highly specific and sensitive to the presence and abundance of individual isotopes. These include ^235^U and ^239^Pu in highly enriched uranium and WGPu^12^. Also unlike NRF, the resonant absorption of epithermal neutrons in the beam can be observed directly with very high resolution (less than eV). This can be done by using time-of-flight (TOF) techniques, as described in detail in Supplementary Note 1. These characteristics allow for direct measurements of resonant absorption, thus enabling ZK implementations otherwise impossible with NRF.

## Results

### Template verification protocol

The key to any verification procedure is a protocol that can guarantee that no treaty accountable item undergoing verification is secretly modified or replaced with another object. Significant thinking has been invested into the concept of template verification, particularly at the US national laboratories and think tanks^4,13,14^. The high-level protocol has been either outlined in or been the basis of prior warhead verification publications in academia^5–8^ and in the US national laboratories^14–17^. Its basic steps can be summarized as follows:

1. The inspection party (inspectors) makes an unannounced visit to an ICBM site and randomly chooses a warhead from one of the missiles. The warhead enters the joint custody of the inspectors and the host country (hosts), and can be treated as the authentic template to which all future candidate warheads undergoing dismantlement and disposition will be compared. The warheads may be tagged with a unique measurement device to reduce the risk of diversions, as described by Fuller^4^.
2. The template is transported under the joint custody of the hosts and inspectors to the site where the candidate warheads (henceforth referred to as candidates) will undergo dismantlement, verification, and disposition. To increase the certainty that the template hasn’t been tampered with it could be isotopically taged, for example, using the nuclear material identification system^18^.
3. Both the template and the candidate undergo dismantlement by the hosts in an environment that cannot be observed directly by the inspectors, but from where no new objects can be introduced to or removed from. The fissile components of the weapons, also known as the pits, are extracted. Each pit is placed in a marked, opaque boxe. The remaining components are placed in a different, unmarked box.
4. The dismantlement area is made accessible to the inspectors. The inspectors use a simple Geiger counter to verify that the non-fissile, unmarked boxes do not contain any radioactive materials, thus confirming that the marked boxes indeed contain the pits.
5. The two marked boxes undergo epithermal transmission analysis. See Supplementary Note 3 for a discussion on aligning the objects while maintaining secrecy. The two-dimensional (2D) radiographs and the spectral signatures are compared in a statistical test. An agreement confirms that the candidate is identical to the template and thus can be treated as authentic. A disagreement indicates a hoaxing attempt.

The last step is key to the whole verification process, and is the focus of this work. The transmission measurement produces an effective image in a 2D pixel array made of scintillators that are sensitive to epithermal neutrons. Furthermore, by choosing a detector with a fast response time and knowing the time when the neutrons are produced, a TOF technique can allow an explicit determination of neutron energies.

### Epithermal neutrons

**Fig. 1:**
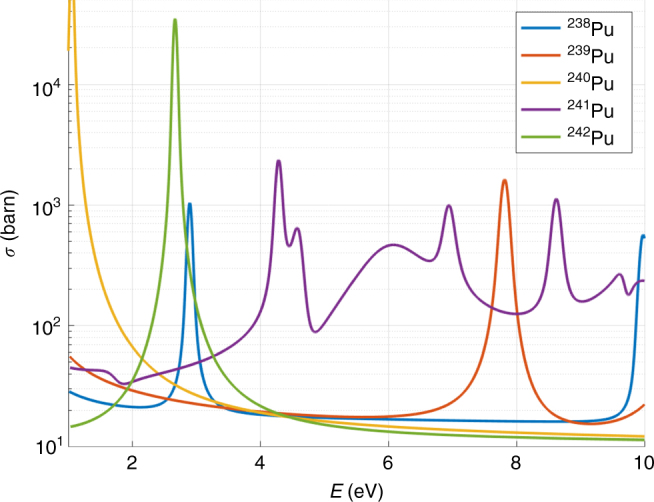
Epithermal neutron interaction cross sections for plutonium isotopes. The legend lists the five isotopes of interest. For reference, WGPu is almost entirely made out of ^239^Pu and ^240^Pu, while RGPu contains significant contributions from ^240,241,242,238^Pu. Evaluated data taken from the JEFF-3.2 database^28^

The epithermal range refers to the neutron energy domain encompassed between the thermal energies of ~40 meV and fast neutron energies of ~100 keV. The energies of interest for this study are those of 1 ≤ *E* ≤ 10 eV. While the neutron interactions in the thermal regime are described by monotonic changes in cross sections, in the epithermal range the neutrons can trigger various resonant responses in uranium and plutonium. These are typically (n,fission) or (n,*γ*) reactions, resulting in the loss of the original neutron. A plot of total interaction cross sections in the epithermal range for five isotopes of interest can be seen in Fig. 1. For a radiographic configuration these interactions selectively remove the original neutrons of resonant energies from the transmitted beam and give rise to an absorption spectrum, resulting in unique sets of ~0.3 eV wide notches specific to each isotope. While the resonances are the most prominent features of the cross section, the continuum between the resonances also encodes information about the isotopic compositions of the target. These combined absorption features yield a unique fingerprint of a particular configuration of isotopics, geometry, and density distribution.

**Fig. 2:**
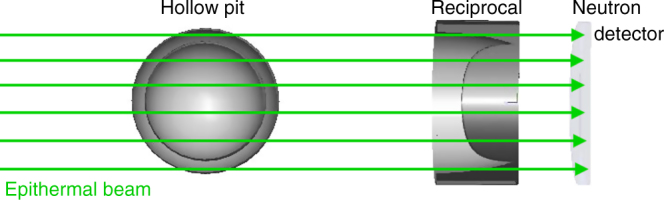
A diagram of the pit and its reciprocal mask in a radiographic configuration. The combined transmission image will be identical to that of a flat plate with a thickness equal to the external thickness of the mask

The key component of a nuclear weapon is its pit—the hollow plutonium sphere at the center of the assembly. This work focuses on the verification of the authenticity of the pit. A direct transmission imaging of the pit would reveal many of its secret parameters. Thus, an additional physical cryptographic barrier is necessary to achieve an ZK test. The barrier needs to be constructed such as to also allow for pit-to-pit comparisons that can detect any significant differences. These two simultaneous goals can be accomplished by the mass reciprocal mask of the pit. The reciprocal mask has a shape such that all the rays of epithermal neutrons in the beam transit the same combined areal density. A simple example of a reciprocal mask of a hollow shell could be a cube with the geometry of the said hollow shell subtracted. Thus, the aligned combination of the pit and the reciprocal will result in uniform areal density, producing an image in the detection plane, which is consistent with that of a flat object. A more optimal reciprocal for a hollow shell of internal and external radii *r*_1_ and *r*_0_ can be defined via its thickness along the beam axis $d = D - 2\left( {\sqrt {r_0^2 - y^2} - \sqrt {r_1^2 - y^2} } \right)$ for *y* < *r*_1_ and $d = D - 2\sqrt {r_0^2 - y^2}$ for *r*_1_ ≤ *y* ≤ *r*_0_, where *D* is the combined thickness observed by all particles in the beam, and *y* is the vertical coordinate. A combination of the pit and the reciprocal is illustrated in Fig. 2. The hosts can fit the pit and the reciprocal inside two opaque boxes, thus keeping their shape secret during times when they are accessible to the inspectors. The two boxes can then be aligned with each other, thus aligning the pit and the reciprocal for the transmission measurement. For a detailed discussion on the reciprocal geometries, as well as details on alignments see Supplementary Note 7. It should be noted that an assembly with a uniform areal density does not mean that the resulting image will be flat as well. Secondary processes, for example, neutron scattering, can distort the image and introduce some angular dependencies that hypothetically could contain information about geometric structures. For this reason detailed simulations are necessary to validate the concept and demonstrate information security. In the next sections we use Monte Carlo (MC) simulations to show that the transmission image produced for this configuration is identical to that of a uniform plate. This outcome guarantees that no geometric information is revealed by the transmission analysis. Even if the inspectors are capable of determining the incident flux in the neutron beam they will at most gain knowledge of an upper limit on the amount of total mass. The hosts can modify the mask to make that knowledge of no value: for example, the inspectors might determine that the mass of WGPu in the pit is <10 kg—this knowledge is useless, as the critical mass for a WGPu pit is approximately 6 kg.

### Isotopic hoax resistance

As the inspectors perform the radiographic interrogation of the pit, they need to ascertain that the isotopics and effective densities of the template and the candidate pits are identical. At a given pit orientation, this can be performed by comparing the epithermal spectra of the transmitted beam from the template and candidate measurements. The energy information can be acquired from the timing of the arrival of the neutrons, via the previously described TOF method to very high precision with either boron-doped microchannel plate detectors or ^6^Li-based LiCaAlF_6_ scintillators^19,20^. For a further discussion see Supplementary Note 9. To study the system’s sensitivity to hoaxing, we consider a scenario where the WGPu pit has been replaced with a RGPu pit. MC simulations are performed for the configuration described in Fig. 2. The simulations were performed using the MCNP5 package, which has a fully validated neutron physics module^21^. The neutron energies were uniformly sampled in the 0 ≤ *E* ≤ 10 eV range. For this study the isotopic mass concentrations for RGPu was 40.8% ^239^Pu. For WGPu the fractions are 93% ^239^Pu, the remainder as ^240^Pu and trace amounts of the other isotopes. See Supplementary Note 5 for detailed listing of the isotopic concentration. For both the template and the hoax RGPu pit the inner diameter was 6.27 cm and the outer diameter was 6.7 cm—based on public domain estimates of a Soviet tactical thermonuclear warhead pit geometries^22^. The reciprocal was modeled in three dimensions along the previously described formula. The combined pit-reciprocal thickness was 5 cm.

**Fig. 3:**
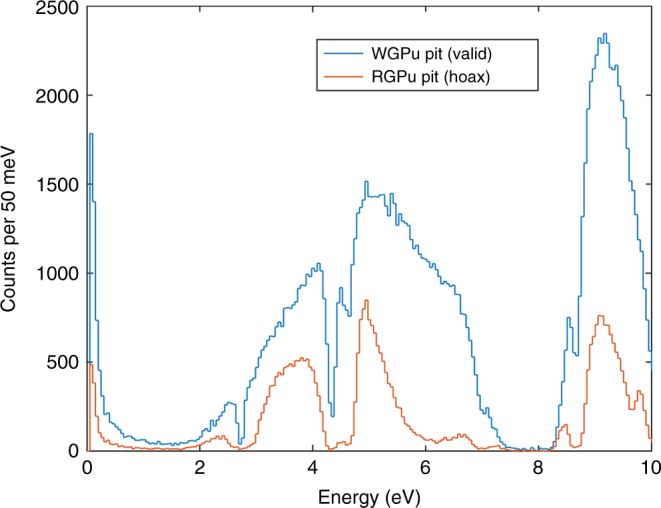
Spectra from transmitted epithermal flux simulations. The simulations were performed for 20.7 × 10^6^ neutrons incident upon a valid WPGu-reciprocal and hoax RGPu-reciprocal configurations

Figure 3 shows the results of the MC simulation of an idealized detector exposed to the transmitted flux through a combination or a reciprocal mask and a WGPu pit, and a similar configuration where WGPu has been replaced by RGPu. The large spectral discrepancies are clear. The difference is primarily caused by the abundance of ^242^Pu in the RGPu, produced as a results of neutron capture in the reactor core. This manifests itself in deeper absorption lines at 4.2 and 8.5 eV, as well as generally increased absorption in the 5–8 eV range. It should be clarified that both the shape and the magnitude of the distribution are part of the hoax sensitivity. To achieve an absolute magnitude measurement, the inspectors can normalize the output to a parameter proportional to the incident neutron flux, such as the measurement time or the beam charge, as appropriate. The results have also been used to determine the number of incident neutrons to achieve a discrimination with a 5*σ* confidence. That number is *n* = 1 × 10^5^ neutrons. See Methods for more detail.

### Geometric hoax resistance

**Fig. 4:**
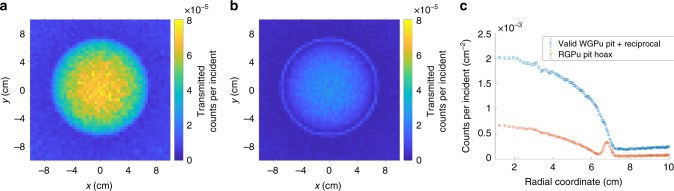
Epithermal neutron images in the detector for two configurations. **a** The valid WGPu-reciprocal scenario, **b** the RGPu-reciprocal hoax scenario, and **c** the radial distribution of the counts for both scenarios showing a clear discrepancy. The non-flat image of the WGPu configuration is caused by the in-scatter of the epithermal neutrons. The error bars reflect one sigma standard deviations

Geometric hoax resistance implies the use of 2D imaging to identify any significant geometric and/or isotopic differences between the template and the candidate, as a way of detecting cheating attempts. The detector described in Fig. 2 and modeled in the MC simulation, whose spectral output is shown in Fig. 3, can be pixelated to produce imaging data. Figure 4 shows the results of simulated epithermal transmission images for a WGPu template and RGPu hoax candidate scenarios. The 2D images show a clear difference, indicating that the candidate is a hoax and thus confirming the isotopic analysis described earlier. The one-dimensional projection of the two images’ radial distributions further shows the extent of the discrepancy. It should be noted that the non-flat image of the WGPu configuration is caused by the in-scatter of the epithermal neutrons, and doesn’t reveal any information about the object, as will be shown in the section on geometric information security. The imaging analysis described here focuses on a hoax scenario of modified isotopics. However, any changes in pit diameter(s) and/or density will result in nonuniformity in areal density as observed by the incident epithermal beam and will thus cause imaging inconsistencies similar to the ones described in Fig. 4.

While the above discussion is indicative of the strength of the technique in detecting hoaxes, a proof of uniqueness is necessary. This means showing that for a given reciprocal a given image can be produced only by a unique object. This is not the case for a single projection, since transmission is only sensitive to the line integral along the beam axis. To exclude the possibility of geometric hoaxes with identical line integrals but consisting of a different three-dimensional manifold, multiple projections along the *x*-, *y*-, and *z*-axes may be necessary. For a spherically symmetric pit similar to the one treated in this work this could be achieved by multiple measurements at random angles. For a spherically non-symmetric objects three projections along the *x*-, *y*-, and *z*-axes may be sufficient—each requiring a reciprocal built for that projection. A more rigorous proof of uniqueness is part of future work, and could be based on the methodology of *K*-transforms as described in Kemp et al.^8^.

### Geometric information security

Geometric information security refers to the notion that the inspectors will learn nothing about the pit geometry beyond what they already know. We assume that for the proof system to be ZK we need to only show that for an honest pit the combined pit-reciprocal transmission is identical to that of a flat, uniform plate of no geometric structure. This implies that the information content in the data cannot allow the inspectors to distinguish from a complex geometry (pit-reciprocal) and a flat object.

**Fig. 5:**
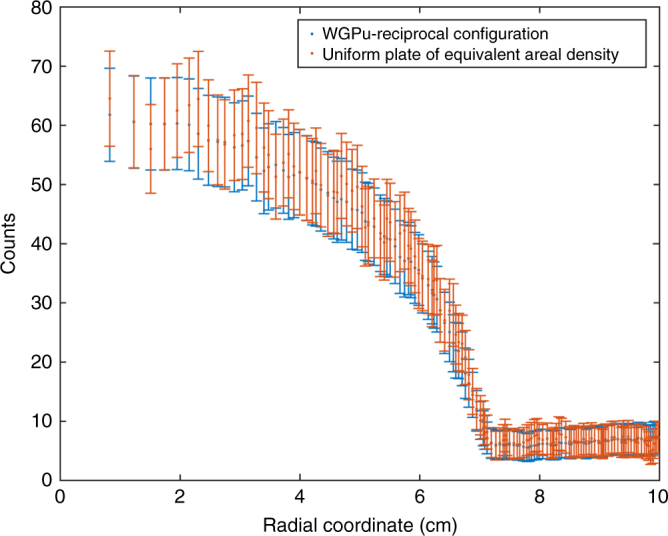
The radial distribution of epithermal neutron counts. The blue markers refer to the pit-reciprocal configuration, while the red markers refer to a flat plate of WGPu of equivalent thickness. The error bars reflect the expected one standard deviation fluctuations for a measurement necessary for the detection of a hoax at a 5*σ* confidence level. The comparison shows statistically identical distributions, indicating that no information about the pit-reciprocal geometry can be extracted

To show this, MCNP5 simulations have been performed for a simple flat plate of WGPu with an areal density equal to that of the pit-reciprocal configuration shown in Fig. 3. Similar to the analysis performed in the section on geometric hoax resistance the radial distribution of the counts of the two configurations are plotted in Fig. 5. The radial count distribution has error bars, which reflect the fluctuations that an inspector would observe for the scenario of 1 × 10^5^ incident neutrons necessary for achieving a hoax detection at the 5*σ* confidence level. The large overlap of the errors shows the statistical identity of the two outcomes, and indicates that no useful geometric information can be extracted about the object.

### Isotopic information security

The isotopic composition of the plutonium pit can affect the reliability of the nuclear weapon^23^. Additionally, the knowledge about the abundance of particular isotopes can allow an observer to determine the methodology by which the fissile material was produced. Because of this and other considerations the information on isotopic composition can be of sensitive nature. Thus, a ZK proof system should not produce any data from which the isotopics of the pit-reciprocal configuration can be inferred beyond what is commonly known.

**Fig. 6:**
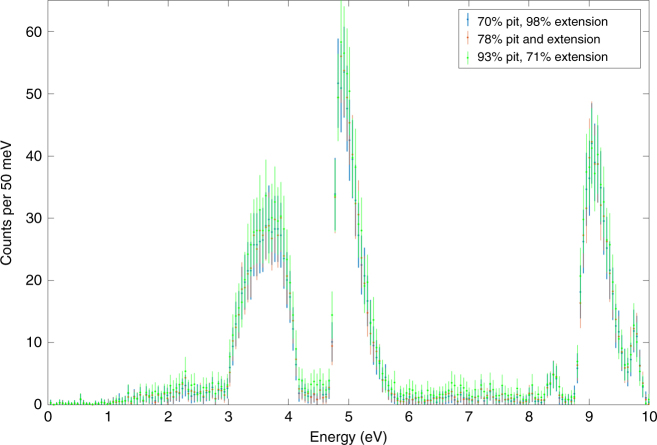
Simulations of spectra of the transmitted epithermal neutron count mean values. The target consisted of a pit-reciprocal and an extension of various levels of ^239^Pu enrichment. The results show that the data would make it impossible to determine the enrichment of the pit beyond the 70–93% range. The error bars reflect the statistical uncertainty of an expected measurement with 1.0 × 10^7^ incident neutrons, which is more than sufficient for achieving the 5*σ* hoax detection requirement

Just as the shape of the reciprocal mask protects the geometry of the pit, its isotopic composition can be used to mask the real isotopic concentrations in the pit itself. While the inspectors may use the spectral information to infer the isotopic ratios for the pit-reciprocal combination, it can be made impossible for them to infer the isotopic contributions of the pit itself. For a simple case consider a slightly modified version of the reciprocal than the one presented in Fig. 2, where the host has added an additional flat, cylindrical extension of unknown thickness and isotopic composition. It can be shown that multiple combinations of isotopics in the pit-reciprocal and the extension will produce the same spectrum. To show this computationally, MCNP5 simulations have been performed for the following three configurations: pit-reciprocal made out of WGPu, and a 2 cm extension of 41% enriched RGPu; pit-reciprocal and extension at intermediate 78% enrichment; and pit-reciprocal at low-intermediate 70% enrichment and extension made of super-grade plutonium. See Supplementary Note 5 for the full listing of concentrations corresponding to different enrichment levels used in these simulations. In the simulation incident epithermal neutron events were uniformly sampled in the [0,10 eV] range. The results of the simulations were used to determine the expected detector counts for achieving a 5*σ* hoax detection. The mean expected counts and the corresponding statistical errors are plotted in Fig. 6. The plots show no statistically significant differences. This proves that for this particular extension thickness the inspectors cannot determine the enrichment level to better than the 70–93% range. The function of the extension is somewhat analogous to that of the encryption/witness foil in tNRF-based warhead verification^8^. Just as the foil scrambles the isotopic-dependent signal, the extensions modify the signal in a way that is fixed between measurements but unknown to the inspector.

It can be shown that the range of uncertainty on the isotopic vector can be determined from ${\mathrm{\Delta }}{\bf{r}} = \frac{y}{x}\left( {{\bf{r}}_{{\mathrm{max}}} - {\bf{r}}_{{\mathrm{min}}}} \right)$, where *x* and *y* are the pit-reciprocal and extension thicknesses, respectively, and **r**_min_ and **r**_max_ are vectors of the lowest and highest possible enrichment levels. This range can be widened arbitrarily by using an extension of lower ^239^Pu concentration or of higher thickness. For a more general mathematical treatment of isotopic information security, as well as a discussion on the possible use of double-chopper and velocity selector techniques for further information protection, see Supplementary Note 6.

## Discussion

Nuclear arm reduction treaties have long suffered from the lack of a reliable, hoax-proof, and information-secure methodology for the verification of dismantlement and disposition of nuclear weapons and their components. The work presented here covers the basic concept behind an epithermal ZK verification system, which targets the fissile component of the weapon. The MC simulations show that epithermal neutrons can be used as a basis of an ZK proof system, allowing to authenticate a fissionable object, such as a hollow sphere, by comparing it against another, previously authenticated template. As required by the ZK proof, the data are physically encrypted, meaning that all the encryption happens in the physical domain prior to measurement, making it impossible for the inspection side to infer significant information about object enrichment and/or geometries. This work has shown that the technique can be made simultaneously hoax-resistant and information-secure in isotopic and geometric domains. While information security implies inability to infer unique parameters about the pit from a pit-reciprocal measurement (due to an unknown reciprocal composition), the hoax resistance implies ability to observe different signals from different objects (due to fixed reciprocal composition). The measurement times, using established techniques for producing pulsed epithermal neutrons, could be made <1 s.

Significant additional research is needed for further understanding the strengths and limitations of this technique. A more rigorous treatment of the geometric aspects of the proof system is necessary, to determine and quantify the resistance against possible geometric hoaxes. This includes the task of showing that a specific measurement is unique to an object of specific isotopics and geometric shape. In this context only hoax objects valuable from a manufacturing standpoint are of importance. The proof of uniqueness in the formalism of *K*-transform^8^ applied to the problem of an epithermal conical beam holds promise.

Future research should also focus on a physical proof of concept implementation of this methodology. A source of epithermal neutrons, either using a research reactor- or an accelerator-based nuclear reaction, can be used as a platform for such an implementation. Particle detection techniques based on existing epithermal neutron detectors should also be researched and optimized^24^. The impact of of object-to-object variability on information security and specificity of this verification system should also be analyzed.

Another important consideration is the actual high-level protocol: it should be recognized that any uncertainty about the protocol is transferred to the whole verification process. The template selection and authenticity is thus critical and should be the focus of additional research.

Finally, the ZK verification technique can also be extended to weapon components made out of low-Z elements. Most hydrogenous materials, for example, explosive lenses, are essentially opaque to epithermal neutrons, thus necessitating the use of other, more penetrating particles. The fast neutrons at MeV scale, where interaction cross sections for hydrogen are significantly lower, are a viable alternative for a source. An established technique of fast neutron resonance radiography, which exploits the resonances in nitrogen, oxygen, and carbon at the ~MeV scale, could prove promising^25^. Other neutron interrogation techniques are also possible. For example, the nuclear material identification system^18^ uses active measurements of neutron multiplicities to achieve a unique photon–neutron tag of a fissile object. This tag depends on the isotopics of the object and its geometry. In its simplest form the technique is not ZK—however, developments of schemes to protect information are possible and could be researched.

## Methods

### Epithermal neutrons

The resonant effect described above has been used in the past for non-destructive assay of nuclear fuel in a technique known as neutron resonance transmission analysis (NRTA). In particular, experiments at Los Alamos National Laboratory’s Lujan Center focused on imaging of fresh fuel samples, while distinguishing between various fissionable isotopes and tantalum impurities^24^. The experimental data were compared to simulations, showing a clear agreement and thus validating the nuclear data, which are also the basis of the MCNP5 simulations in this work. Furthermore, work at Idaho National Laboratory proved the feasibility of using NRTA for assaying plutonium in fuel^12^. These and other results (a) demonstrate the feasibility of using epithermal neutron beams for imaging and assaying fissionable materials and (b) validate the input parameters and cross sections in the MC used in this work. It should be pointed out that while some minor inaccuracies in the cross sections are possible, the measurements described in this work are differential—thus are less sensitive to absolute systematic uncertainties.

### MC simulations and statistical test

For the isotopic hoax resistance simulation 20.7 × 10^6^ neutrons were uniformly sampled in the 0–10 eV range, and the energy of the output was reported in a histogram of 202 bins. For two given spectra, the chi-square test can be applied to reject or accept the null hypothesis, i.e., the hypothesis that the fluctuations are merely statistical and normally distributed. For this result the value is *χ*^2^ = 67 177, translating to a confidence for rejecting the null hypothesis of essentially exactly *p* = 1.0 and showing conclusively that a hoaxing attempt is underway. Furthermore, it is possible to determine the minimum number of incident epithermal neutrons necessary for achieving a confidence of *p* = 1–2.9 × 10^−7^, which corresponds to the standard 5*σ* test. That number is *n* = 1 × 10^5^ neutrons. For comparison, the MIT research reactor has been used to produce 10^10^ s^−1^ cm^−2^ epithermal neutrons in the [1 eV,10 keV] range^26^. For this beam it corresponds to ~10^9^ s^−1^ in the [1,10] eV range. Thus, a measurement requiring 10^5^ neutrons would require fractions of a second. Alternative and perhaps simpler sources of epithermal neutrons are also possible, for example, using compact, 2 MeV proton cyclotrons and ^7^Li(p,n)^7^Be reactions^27^. Such sources would produce enough epithermal neutrons to achieve necessary measurements in about 12 s. Other sources of epithermal neutrons are spallation sources using higher-energy proton accelerators, as described in Losko et al.^24^. See Supplementary Note 2 for additional discussion and detailed calculations.

### Data availability

The data that support the plots within this article and other findings of this study are available from the corresponding authors on reasonable request.

## Electronic supplementary material

Supplementary Information(PDF 872 kb)
