# Supplementary material for: Nuclear disarmament verification via resonant phenomena
Source: Nat Commun. 2018 Mar 28;9:1259. doi: 10.1038/s41467-018-03680-4 (PMC5871754; doi:10.1038/s41467-018-03680-4)
Supplement: Supplementary file 1 — Supplementary Information(PDF 872 kb) [file 41467_2018_3680_MOESM1_ESM.pdf]

Supplementary Note 1

TOF methods. The technique described in this work requires one to determine the energy of every neutron count in the detector. For the neutrons in the cold, thermal, and epithermal range this can be achieved via a pulsed source and time-of-flight (TOF) technique. If the neutron pulse occurs at time t_0, and the non-relativistic neutron is detected at time $t = t_0 + \Delta t$ at a distance d then its energy is

$$E = m\frac{(l/\Delta t)^2}{2} \tag{1}$$

where m is the neutron mass and $\Delta t = t - t_0$ is TOF. By propagating the errors we can determine the uncertainty in E: $\delta E = \delta\Delta t m l^2/\Delta t^3$. The uncertainty in Δt primarily comes from that of t_0, since most scintillation and microchannel based detectors have extremely high rise times. Here the uncertainty on t_0 is either the opening time of the chopper, or the pulse length of the accelerator that produces the epithermal neutrons via nuclear reactions. Thus, we can write

$$\frac{\delta E}{E} = 2\frac{\delta t_0}{\Delta t}. \tag{2}$$

Taking 5 eV as a point midway in our energy range, it is possible to determine the maximum time-width of the pulse to achieve the energy resolution of $\delta E =$ 0.3 eV at the distance of $l =$5 m: $\delta t_0 = \frac{\Delta t}{2}\frac{\delta E}{E}$. For this distance $\Delta t = 161\mu$s, and thus $\delta t_0 = 5\mu$s. The precision of energy reconstruction can be increased by either making the pulse shorter, or by moving the detector further away and thus increasing Δt. Since the geometric acceptance changes quadratically with l,

it is statistically more optimal to shorten the pulse length than to lengthen the distance by the same fraction.

The TOF technique simply needs a neutron beam in a pulsed mode. In Supplementary Note 2 a few methods for such beams are described. For the platforms which use a DC neutron source, for example a reactor or a moderated isotopic neutron source, a chopper is necessary for producing a neutron pulse with a well defined t_0. For most thermal beams cadmium is the preferred material, due to the $\sim 10^4$ barns cross section of ^{113}Cd isotope. For the epithermal range of $\sim$5eV however the cross sections drop to just a few barns. A much better material for stopping the epithermal neutrons are boron and lithium. ^{10}B in particular, which has 25% abundance, has a cross section of approximately 250 barns. A boron chopper of thickness of just 3 mm will attenuate the neutrons by a factor of $\times 10^6$.

The chopper needs to be rotated at a frequency such that the opening time of its slot is $\leq 5\mu$s, as determined above. Thus, a chopper could be a disk of radius of 50cm, made of 3mm of boron carbide sandwiched between two disks of steel of identical radius, for mechanical stability. If rotated at the frequency of 3600 rpm, a slit of 0.5 mm will yield an opening time of $2.7\ \mu$s. The chopper may have ten such equidistant slits, to recover some of the duty factor. Other possibilities may involve the use of turbomolecular pumps, which can easily achieve rotational speeds of 90000 rpm, with a narrow hole drilled through the boron-coated rotor blades. The neutral particle analyzer, built and installed at MIT's Alcator C-mod tokamak, could be an example of such a chopper[1].

Supplementary Note 2

Epithermal neutron production by nuclear reactions and nuclear reactors. The nuclear research reactors are used as very intense sources of neutrons. Depending on the configuration, the output neutron beam's energy distribution can be thermal, epithermal, or fast. The MIT reactor has been used to produce epithermal neutron beams for oncological applications[2]. Using a fission plate converter, beams of 10^{10} s^{-1}cm^{-2} in the $[1eV, 10keV]$ range have been achieved. The object in our study has a radius of 7 cm, thus the total neutron flux in the $[1, 10]$ eV range will be $\sim 10^9$ s^{-1}. This flux however will have to be modified using a chopper, in order to enable energy reconstruction via the above-described TOF techniques. With a chopper opening of 5 μs, and a distance which corresponds to $\Delta t = 161$ μs, the chopper will have a maximum duty of 3%. However, the presence of wraparound events, i.e. thermal neutrons with arrival times of $n \cdot 161$ μs, where n is an integer > 1, can introduce uncertainties in energy reconstruction from TOF. This can introduce significant backgrounds. To avoid this, the chopper can be kept closed for $4830\mu s$ - this will eliminate all thermal neutrons down to the energy of 6 meV, while reducing the chopper duty to 0.1%. Combining these numbers, the total epithermal flux of neutrons in the energy range of $[1, 10]$ eV will be 10^6 s^{-1}. With only 10^5 neutrons needed for the configuration described in the main body, this translates to a measurement time of 0.1 second. Another possible source of epithermal neutrons is a neutron isotopic source, such as a ^{252}Cf source of high intensity, moderated with polyethylene or water, thus shifting the fast 1MeV neutrons to the epithermal regime.

The epithermal neutrons can also be produced using nuclear reactions between accelerated

light ions and various targets. There are two classes of light ion based nuclear reactions that can produce neutrons in the epithermal range. Significant work using epithermal neutrons was performed using the Los Alamos National Laboratory's 800 MeV proton spallation source, which produces neutrons of a broad range of energies[3]. This source was the basis of a number of beam lines used for a range of applied and fundamental studies. These included epithermal beams used for the non-destructive assay of nuclear fuels[4]. However a particularly attractive and compact alternative to a large spallation facility are smaller proton accelerators, which allow to trigger the ^{7}Li(p,n)^{7}Be and ^{9}Be(p,n)^{9}B reactions. A careful operation of incident proton energies in the initial state and neutron angles in the final state can allow to create adequate intensities of epithermal neutrons. Herrera et al.[5] provide calculations and data of the dependence of the double differential neutron yield on incident proton energy and emitted neutron angle.

Supplementary Figure 1 shows the double differential yield, plotted against emitted neutron energy and angle, as well as plotted against neutron energy in the $[0, 10]$ eV range for the emission angle of 90°. Using the 1 eV^{-1}sr^{-1}μC^{-1} as the order-of-magnitude value and assuming a beam opening of 30° the expected total epithermal yield in the $[0, 10]$eV energy range incident upon the target will be $r = 8500$ s^{-1}mA^{-1}. Some off-the-shelf commercial accelerators can produce 2 MeV proton beam currents of ~ 50 mA[6,7]. It was determined that about 10^5 incident neutrons are necessary for achieving rejection of hoaxes at the 5σ confidence level. Taking a proton beam current of 1 mA, this translates to a measurement time of just 12 seconds.

While the ^{7}Li(p,n)^{7}Be is an attractive reaction, a number of other, similar reactions exist,

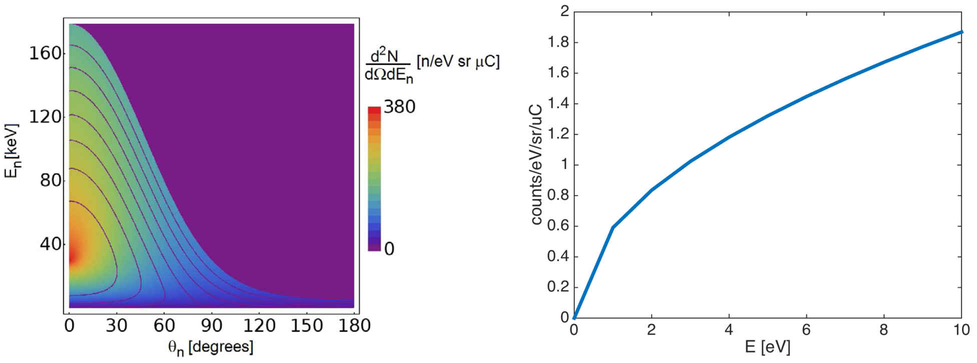

(a) **(b)**

Supplementary Figure 1: Neutron yields. **a.** double differential neutron yield (colors) as function of emission angle and neutron energy for different incident proton energies on natural thick lithium target. Higher values of the incident proton beam energy broaden the accessible region. Here energies of (1.89+0.01n) MeV contours are plotted. From Herrera et al.[5]. **b.** epithermal neutron yields for a natural lithium target and a 2 MeV proton beam, computed for the emission angle of 90°. Reprinted from Nuclear Instrumentation and Methods in Physics Research Section B, 349, Herrera, M.S. et al., "New methods to evaluate the 7Li(p,n)7Be reaction near threshold," pages 64-71 (2015), with permission from Elsevier.

such as ^{9}Be(p,n)^{9}B. Some of these may have higher epithermal neutron yields. The search for an optimal reaction is outside of the scope of this work and may be a subject of future studies.

A significant challenge when using TOF techniques is the presence of the thermal neutrons, which arrive at times much longer than the waiting time for the epithermal pulse, thus making it difficult or impossible to identify the originating pulse. This could introduce uncertainties in the TOF reconstruction. However, the calculations shown in Supplementary Figure 1 show that

the (p, n) reactions have almost no thermal flux, thus significantly limiting their impact on the precision of the TOF method.

Supplementary Note 3

Alignment, variations in design, and other systematic effects. In order to perform the verification measurement in a manner which is information secure, the hosts need to align the objects - the template/candidate with the reciprocal - in a way that the inspectors cannot observe the objects. At the same time the inspectors need to be able to confirm visually that the boxes containing the template and candidate were switched between the two measurements. To achieve these dual goals the hosts need to place the three objects in three opaque boxes while aligning the objects with fiducial marks on the external surfaces of the box. Then, after the boxes are closed the inspectors can visually access the area. At this stage the template-reciprocal alignment can be performed using the fiducial marks. After the first measurements the template/candidate boxes are switched, and a new candidate-reciprocal alignment is performed. In this process the inspectors will not be able to observe the objects directly, the hosts will be able to perform the object alignments via the external fiducials, and the inspectors will be able to visually confirm that the template and the candidate have been switched between measurements.

Two issues come to the fore when discussing the use of reciprocals for a zero-knowledge proof system. As in any detection system, this verification system's sensitivity will have its limits, affecting the inspector's ability to distinguish between objects of various sizes or different isotopic

concentrations. The detection probability of the system is determined by measurement times, detector sensitivities, as well as the specificity of neutron interaction physics. These factors are mostly of stochastic nature, and measurements of arbitrary sensitivity can theoretically be achieved by varying the measurement times. However, systematic effects are also present. These include the unit-to-unit variability, due to manufacturing precision, as well as the hosts' ability to align the template and the candidate with the reciprocal. Modern surveying methods allow alignment precision down to the $\mathcal{O}(10\mu m)$ scale, thus the main difficulty is related to the actual unit-to-unit variability.

Information on manufacturing precision are not available in open domain. For a given variability, the two sides can agree to broadened criteria of verification in order to accommodate such variability and thus avoid false alarms and reveal information about the geometry of the pit.

This circumstance in its turn limits one's ability to achieve arbitrary sensitivity. It is reasonable to assume that manufacturing variations are small, and thus the hoaxing scenarios attainable due to this limit on sensitivity are probably not of a significant advantage to either side in the inspection regime. A more rigorous treatment of this problem should be part of future research, possibly in the classified domain.

Supplementary Note 4

Probabilistic Tests and minimum necessary counts for 5σ detection. For a particular energy bin i, the statistical significance in units of sigma can be determined via $n_i = (c_{0,i} - c_{1,i})/\sqrt{c_{0,i} + c_{1,i}}$,

assuming Poisson statistics, where c_0 and c_1 are the counts from the two distributions undergoing comparison. In frequentist statistical analysis, and assuming normally distributed errors we can determine the probability that the disagreement between $c_{0,i}$ and $c_{1,i}$ is consistent with the null hypothesis, i.e. is caused purely by statistical fluctuations. Conversely, the confidence for rejecting the null hypothesis and accepting the anomaly hypothesis (for example a hoaxing scenario is underway) can be determined via $p_i = 1 - C(0, n_i)$, where $C(0, x)$ is the cumulative distribution. For example, an $n = 5(\sigma)$ outcome (used in high energy physics for identifying new particles) corresponds to a confidence level of $p = 1 - C(0, 5) = 1 - 2.9 \times 10^{-7}$, a very high confidence that can be used as the standard of testing.

For a multi-bin data the more common test is the chi-square test. If the data has N bins, the number of degrees of freedom (NDF) is N. The probability that two distributions are deviating only due to normal fluctuations can be determined from $p = Prob(\chi^2, NDF)$, where $Prob(x, y)$ is the chi-square distribution and χ^2 is the (non-reduced) chi-square that can be computed from $\chi^2 = \sum_i^{NDF} (c_{0,i} - c_{1,i})^2/(c_{0,i} + c_{1,i})$.

For the data presented in Supplementary Figure 3 we have NDF$=202$ and $\chi^2 = 67177$. For this value of NDF the value of χ^2 corresponding to the confidence of $1 - 2.9 \times 10^{-7}$ (the 5σ standard) is just $\chi^2|_{5\sigma} = 319$. Clearly the discrepancy observed here implies an almost complete agreement with the anomaly hypothesis. Furthermore, it is possible to determine the minimum statistics necessary to bring the $\chi^2 = 67177$ result, achieved by using $N = 2.0 \times 10^7$ neutrons, to a 5σ result. Since χ^2 depends linearly on the statistical count, the fraction of statistics necessary

is just $n = N(319/67177)$, i.e. just $n = 1.0 \times 10^5$ incident neutrons. Most epithermal neutron sources can produce this neutron count in a matter of minutes.

Supplementary Note 5

Concentrations of Plutonium isotopes used in Isotopic Information Security Analysis. Supplementary Table 1 lists the concentrations of individual isotopes of plutonium for the various levels of enrichment used in the isotopic information security analysis. For all the objects the density was 19.8 g cm^{-3}.

Supplementary Note 6

Isotopic Information Security. The loss of a beam neutron due to some form of interaction can be described by the attenuation factor $A = \frac{I}{I_0} = \exp(-\mu\rho d)$, where ρ and d are the density and thickness of a medium, $\mu = \sigma N_A/A$ is the mass attenuation coefficient, N_A is Avogadro's number, A is the atomic number and σ is the total energy-dependent interaction cross section. This is only an approximation, because it treats all elastically scattered neutrons as undetected. For a transmission detector with a small acceptance this can nevertheless be a good approximation for an analytical treatment of the dynamics of isotope-dependent transmission.

Consider a particular material with isotopic vector $r_i = \{r_{238}, r_{239}, ..., r_{242}\}$, where individual elements are the fractional concentration of a particular isotope, such that $\sum_i r_i = 1$. Then the

attenuation in a particular energy bin can be determined from

$$A(E) = \exp\{-\rho d \sum_i r_i \mu_i(E)\}. \tag{3}$$

If the object consists of a pit-reciprocal combination of thickness x and a vector r_i, an extension plate (see the main body for an explanation) of thickness y and a vector r'_i, then the logarithm of total attenuation is simply

$$\ln A(E) = -\rho \sum_i \mu_i(E) z_i (x + y) \tag{4}$$

where $z_i = (xr_i + yr'_i)/(x + y)$ is the effective isotopic concentration vector of the combined pit-reciprocal-extension. It can be shown that infinite combinations of x, y, r_i, and r'_i will produce the same value of z_i. To illustrate this consider three scenarios for r_i, r'_i, and $x = 5$cm and $y = 2$cm:

1. Scenario 1: r_i and r'_i correspond to WGPu and RGPu. See Supplementary Table 1 for detailed values. In this case z_i corresponds to intermediate-grade plutonium.

2. Scenario 2: Both r_i and r'_i are simply equal to z_i from Scenario 1, i.e. correspond to intermediate-grade plutonium.

3. Scenario 3: r'_i corresponds to super-grade plutonium. Using z_i from the above scenarios, we find r_i to correspond to low-intermediate grade plutonium.

In these three scenarios the enrichment levels for the pit varied between 70% and 93%, while the effective isotopic concentration vector remained constant at $z_i = \{0.0088, 0.784, 0.1304, 0.0453, 0.0315\}$.

Thus all these scenarios will produce the same transmission spectrum. Supplementary Figure 2 shows the results of calculations of transmitted spectra for these three scenarios, showing identical transmitted outputs.

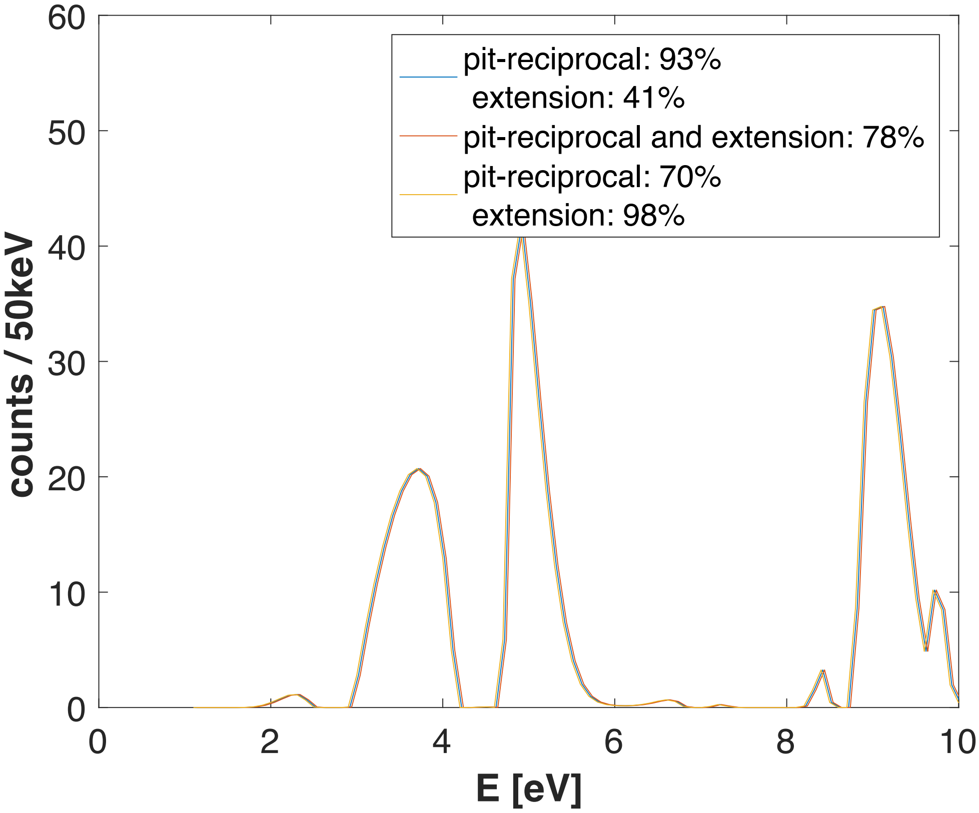

Supplementary Figure 2: Transmitted calculated spectra for Scenarios 1, 2, and 3, for a total of 10mln incident epithermal neutrons uniformly sampled in the 0-10 eV energy range.

In addition to demonstrating this concept via calculations or MC simulations, it is also possible to determine the maximum range of uncertainty for reconstructed r_i, given actual values of $\mathbf{r}$,

$\mathbf{r}'$, and corresponding $\mathbf{z}$. The possible values of $\mathbf{r}$ are limited by

$$\mathbf{r_{min}} = \frac{\mathbf{z}(x+y) - \mathbf{r'_{max}}y}{x}$$
$$\mathbf{r_{max}} = \frac{\mathbf{z}(x+y) - \mathbf{r'_{min}}y}{x}.$$

Here $\mathbf{r'_{max}}$ and $\mathbf{r'_{min}}$ correspond to all possible enrichment levels from which the extension can be made. The maximum is then just the super-grade plutonium, while the minimum can be the reactor grade plutonium. The full range of values of $\mathbf{r}$ is then simply

$$\Delta\mathbf{r} = \mathbf{r_{max}} - \mathbf{r_{min}} = \frac{y}{x}(\mathbf{r'_{max}} - \mathbf{r'_{min}}). \quad (5)$$

By using the values of $x = 5$ cm, $y = 2$ cm, and solving for the ^{239}Pu enrichment r_{239}, we find that the range corresponds to about $\Delta r_{238} = 23\%$, which is consistent with the previous result of 70-93%. This range can be further widened by either increasing y, or using r'_{min} of even lower enrichment.

As already stated, the simple calculation doesn't take into account such effects as in-scatter by neutrons. This necessitates a more thorough MC simulation to fully validate this idea. Such a simulation was performed using the MCNP5 package, and the results can be seen in Supplementary Figure 6 in the main body. The simulations confirm the conclusion of the analytic calculations above.

The importance of the above treatment is great: while the inspectors can use the data to reconstruct z_i, they will not be able to reconstruct r_i beyond simply stating that the pit enrichment level is somewhere between 70% and 93%. The knowledge of this broad range is essentially

useless information, as it is already known that the plutonium in most weapons is at the WGPu enrichment levels. This range can be further broadened, if necessary. As discussed above that can be achieved either by using an extension of lower enrichment level or one of a thicker value of y - albeit at the need for longer measurement times. Finally, the reciprocal mask itself can be made modular: the recessed area shadowing the pit can be made of r_i', while the peripheral part can be made from z_i - thus removing the need for an extension plate.

While the analysis above shows that it is possible to protect the absolute isotopic information, some information about pit-to-pit variability may be inferred by the inspectors from comparative analysis of transmission spectra, for example by observing the variability in the absorption lines due to variable concentrations of ^{241}Pu, which has strong resonances at 4.2 and 8.5 eV. To mitigate this, the hosts and the inspectors could agree to a reduced resolution, as a way of smearing the absorption lines from that particular isotope. There are a few ways of achieving this. One approach would be to broaden the t_0 in the TOF technique by using a broader proton pulse for a ^{7}Li(p,n)^{7}Be reaction[5]. For the case of a chopper technique a wider slit can be used.

If necessary, the information security of the system can be further strengthened by extending the the epithermal neutron source in this proof system with a velocity selection. Velocity selection is a well-established technique for filtering out neutrons based on their energy/velocity. A velocity selector is a system of multiple blades whose length, pitch angle and angular velocity allows only neutrons of a particular velocity range to pass through[10]. A yet simpler configuration would consist of two choppers: the first one setting the t_0, and the second one, with a phase shift, selecting the

neutrons based on their arrival time and thus their energy. Such a device could serve as a physical information barrier, allowing the hosts to limit the measurement to a particular pre-negotiated spectral region(s). Meanwhile the inspector can measure the velocity explicitly via the TOF information, as a way of confirming that the prover is not manipulating the output window of the velocity selector.

Supplementary Note 7

Reciprocal Geometries. The main function of the so-called reciprocal mask is to make it impossible for an observer to extract any sensitive isotopic or geometric information about the pit from a direct transmission measurement of the combined pit-reciprocal geometry. The simplest way of achieving this is by taking a space encompassed by a rectangular prism, filling it with a shape identical to the pit but with the negative of its density, then adding a uniform density until all the negative density voxels have zero density. This amounts to creating the negative of the pit. The 2-d cutaway of such a simple approach can be seen in Supplementary Figure 3.

While intuitively simple, this particular type of reciprocal mask has a number of problems. For example, it would be very hard to keep subcritical. Even if the criticality of the mask can be significantly reduced (for example by slicing it perpendicular to the beam axis and introducing space between the slices), the combined thickness of pit-reciprocal configuration is unnecessarily high, thus necessitating long measurement times for a statistically significant detection.

A much more optimal reciprocal mask can be built simply by realizing that the thickness

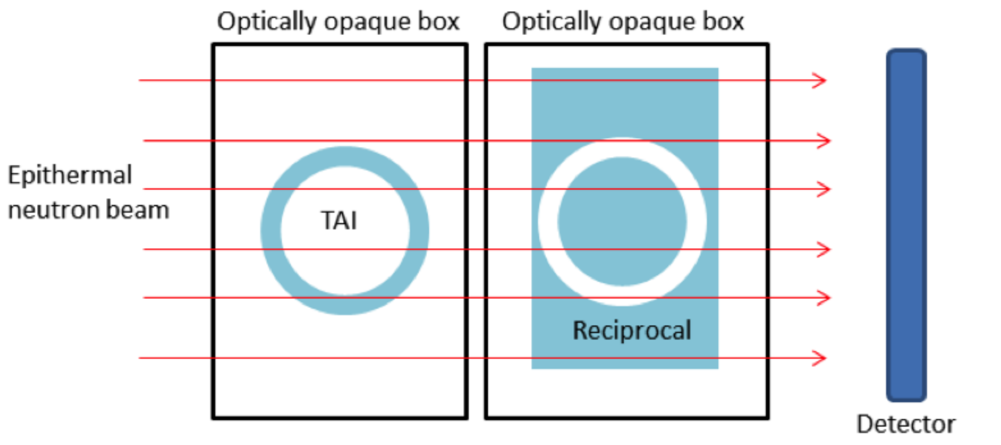

Supplementary Figure 3: The verification concept employs a reciprocal mask which is designed such that the areal-density of a valid pit and reciprocal is uniform when viewed along the beam axis. When radiographed with epithermal neutrons, the resulting detector image is uniform and matches that of a plate of the same areal density. Here TAI (treaty accountable item) refers to the pit. To maintain secrecy, both the pit and the reciprocal should remain at all times in optically-opaque boxes.

of the mask along a transmission axis needs to be equal to $D - z$, where D is some constant combined thickness and z is the thickness of the pit along that axis. So, for a hollow shell of internal and external radii r_1 and r_0 the reciprocal can be defined via its thickness along the beam axis $d = D - 2(\sqrt{r_0^2 - y^2} - \sqrt{r_1^2 - y^2})$ for $y < r_1$ and $d = D - 2\sqrt{r_0^2 - y^2}$ for $r_1 \leq y \leq r_0$, where y is the vertical coordinate. A combination of the pit and the reciprocal is illustrated in Figure 4. For this particular case the combined thickness amounts to $D = 5$ cm.

The geometry and the enrichment of the combined pit and reciprocal is important when it

comes to safety considerations. As suggested earlier, the wrong geometry may either be too close to criticality, or simply impossible to construct. Thus the criticality analysis of the geometries needs to be performed. As a neutron is incident on the pit or the reciprocal, it can trigger neutron induced fission, leading to a fission chain. The time dependence of the chain and the number of fissions can be determined from $N(t) = \exp{(k_{eff} - 1)t/\tau}$, where t is time, τ is the mean lifetime of a neutron in the order of 10 ns, and k_{eff} is the k-effective. Positive values of $k_{\text{eff}} - 1$ cause the reaction to quickly diverge in what is called a criticality event. For example for $k_{\text{eff}} = 1.1$ it would take less than a microsecond for all nuclei in the pit to undergo fission, resulting in a nuclear explosion. On the other hand, for values of $k_{\text{eff}} = 0.9$ the chain will exponentially decay with the lifetime of $\sim$100 ns.

To determine the feasibility and the safety of the proposed configuration a set of MCNP5 simulations were performed to determine the k_{eff}. For a geometry described in Supplementary Figure 4 and made of WGPu the k-effective was determined to be $k_{\text{eff}} = 0.866 \pm 0.001$. A criticality analysis was also performed on the 78% enrichment configuration described in Supplementary Figure 6, where a 78% enriched pit and reciprocal are followed by a 2cm extension of the same enrichment level. For this configuration $k_{\text{eff}} = 0.8318 \pm .0002$. For comparison, the new graphite pile at MIT's Nuclear Reactor Lab has $k_{\text{eff}} \approx 0.82$ (per private communications with Professor Kord Smith, MIT). It is not shielded, is open for general access and for educational purposes, and doesn't require any certification or regulatory oversight. To explore ways of further reducing this number, the reciprocal geometry in Supplementary Figure 4 was modified by breaking it town into individual concentric hollow cylinders, which have been extended along the z-axis in a telescope-

like configuration. Such a modification significantly drops k-effective, bringing it to $k_{\text{eff}} = 0.621 \pm .001$. In conclusion, the assemblies used in the concept described in this work are safe from the point of view of criticality consideration.

Supplementary Note 8

Epithermal neutron backgrounds. The ^{240}Pu isotope of WGPu is known to be a great source of neutrons from spontaneous fission. MCNP5[11] simulations of the neutron emission rates from the hollow sphere described in this work shows that the neutrons are emitted at the rate of 4.5×10^5 s^{-1}. This number includes the fission neutrons and the neutrons from fission chains. A fraction of these neutrons will be in the epithermal regime. A calculation of the fraction of the neutrons in the 1-10 eV range is possible using the Watt formula:

$$F = \int_{10^{-6}}^{10^{-5}} e^{-E/0.965} \sinh\sqrt{2.29E}dE / \int_{0}^{\infty} e^{-E/0.965} \sinh\sqrt{2.29E}dE = 1.4 \times 10^{-8}$$

Thus the number of epithermal neutrons observed by the detector directly from the WGPu assembly will be essentially zero.

However, some of the fast neutrons will scatter from surrounding concrete walls, partially thermalize, and possibly reach the detector in the above mentioned energy range. This is known as room return. Monte Carlo simulations have been performed to estimate the magnitude of this effect. To this end a point source of neutrons has been simulated inside a 8 $\times$ 8 $\times$ 20 m^3 room (for example an experimental hall) with concrete walls, where the neutrons were sampled from the

Watt spectrum. The 10cm $\times$ 10cm detector was placed two meters from the source. The simulation tracked the neutrons until the thermal regime. It showed that only 2×10^{-8} of the fission neutrons will reach the detector in the 1-10 eV range. Based on the above it is possible to determine how many epithermal neutrons will constitute the background in the detector for the worst case scenario of 120 s run time. That number is approximately one neutron.

We thus conclude that the impact of epithermalized fission neutrons from ^{240}Pu on the verification process will be negligible.

Supplementary Note 9

Epithermal Neutron Detectors. The warhead verification process described in this work uses epithermal neutron beams, which, after passing through the objects impinge upon a detector. The detector needs to allow the reconstruction of both the energy and hit coordinate of the incident neutron. The former is achieved via the TOF techniques described in Supplementary Note 1. To this effect the detector needs to have time resolution of less than a microsecond. To achieve a coordinate resolution it needs to be pixelated to a size agreed upon by the treaty parties.

A detector type which achieves both of these goals simultaneously is a ^{10}B doped microchannel plate (MCP), which has been developed by researchers from UC Berkeley and NOVA Scientific, Inc., and is described by Tremsin et al.[12]. The schematic in Supplementary Figure 5 illustrates the basic concept. The incident thermal or epithermal neutron is captured by the ^{10}B doppant in the glass, which leads to the reaction $^{10}B + n \rightarrow {}^{7}Li + \alpha + 2.8MeV$. The 2.8 MeV Q value of

the reaction is shared between the alpha and the lithium ion, which then ionize the channel and start a cascade. The timing resolution of the MCPs is in the order of 100 ps. Comparing this to the $\delta t = 5\mu$s in Supplementary Note 1, we conclude that this timing resolution is abundant for achieving the energy resolution of $\delta E \leq 0.3$ eV via TOF techniques. While the coordinate resolution of the MPCs may be excessively high, its readout may be modified as to sum multiple channel signals together, to achieve the desired resolution.

The ^{10}B doped MPCs have been used in research on fuel sample analysis, using the TOF technique, at Los Alamos Neutron Science Center[4]. Figure 5 and 6 in Losko et al.[4] show isotopic transmission images of uranium samples performed with this technique. Currently the ^{10}B doped MPCs can be commercially acquired from NOVA Scientific, Inc..

While the MPC based imaging of neutrons has been performed in the past, it is by no means the only way of achieving such functionality. Other time and position resolving detectors can be built, for example using low-Z scintillator LiCAF[13]. Cerium-dopped LiCAF scintillator in particular has a very fast time response (<1μs). A hodoscope of LiCAF scintillators, mounted on Silicon photomultipliers (SiPM) can be assembled to achieve both neutron time/energy and hit position resolution. An example of such a configuration can be seen in Totsuka et al.[14]. Such a detector development effort may be part of future research.

	^{238}Pu	^{239}Pu	^{240}Pu	^{241}Pu	^{242}Pu
Super-grade	-	0.98	0.02	-	-
Weapons-grade (WGPu)	0.0005	0.9353	0.0598	0.004	0.0004
Intermediate	0.0088	0.784	0.1304	0.0453	0.0315
Low-intermediate	0.0124	0.7057	0.1745	0.0634	0.0441
Reactor-grade (RGPu)	0.0297	0.4059	0.3069	0.1485	0.1089

Supplementary Table 1: The concentrations of plutonium isotopes for materials used in the study. The concentrations are hypothetical, and are based on Refs.[8,9].

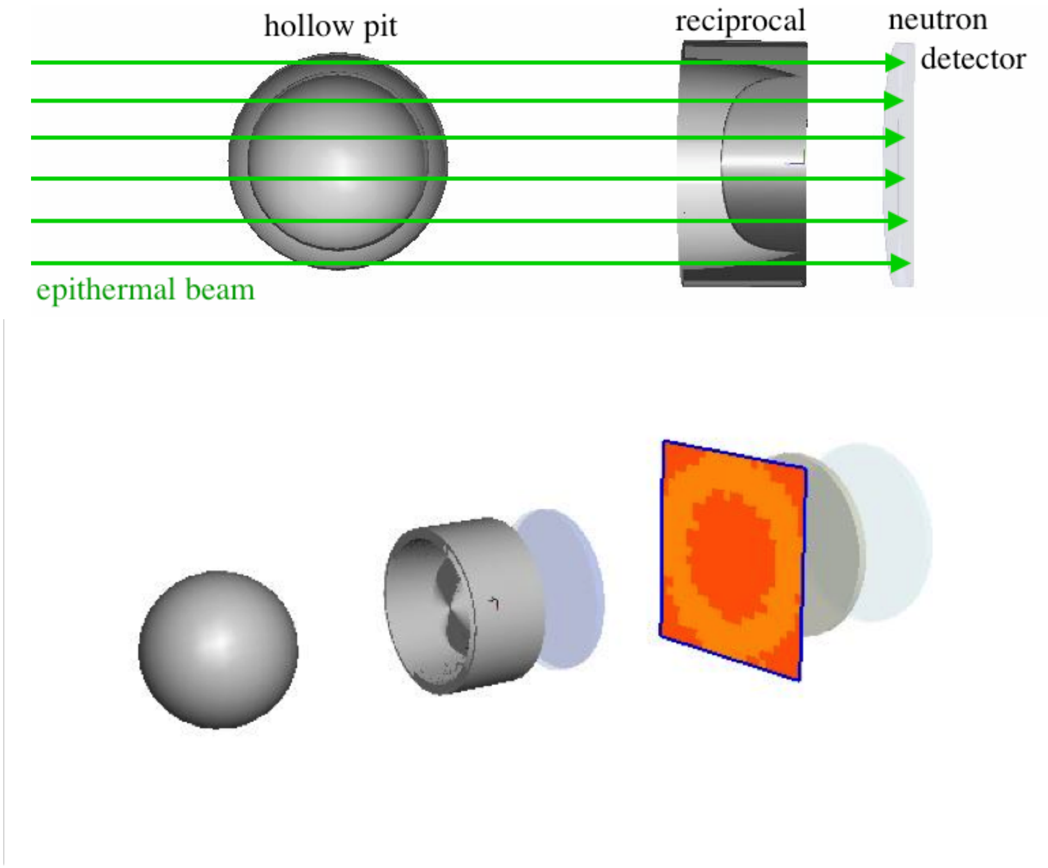

Supplementary Figure 4: Pit-reciprocal configurations. **a.** a diagram of the pit and its reciprocal mask, aligned along the axis of the interrogating beam. The combined transmission image will be identical to that of a flat plate with a thickness equal to the external thickness of the mask. **b.** the 3D view of the pit and the reciprocal.

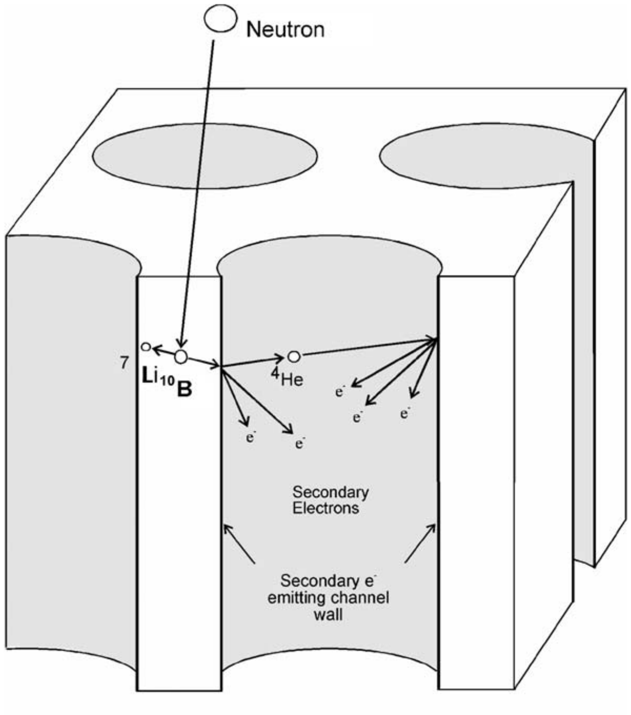

Supplementary Figure 5: Schematic of the operation of a ^{10}B doped MCP neutron imager. From Tremsin et al.[12]. Reprinted from Nuclear Instruments and Methods in Physics Research Section A, Vol. 539, Tremsin, A. et al., "Efficiency optimization of microchannel plate (mcp) neutron imaging detectors. I. Square channels with 10B doping," pages 278-311 (2005), with permission from Elsevier.

Supplementary Note 10

MCNP and Nuclear Data. The work presented herein is entirely based on Monte Carlo simulations. While proof of concept experimentation is necessary for a comprehensive validation of the warhead verification concept presented in this work, the presented MC results are nevertheless a reliable first test of the concept, as they use the MCNP simulation toolkit, which is a well tested and validated package. It should be pointed out that the technique presented in this work is based on differential, rather than absolute measurements. Thus only large inaccuracies in the simulation models can have a significant impact on the feasibility of this warhead verification concept.

MCNP has been extensively used for reactor and weapon design, design of shielding, medical applications, etc. For a report on the validation of the MCNP in the epithermal range see Mosteller et al.[15] (this work refers to the epithermal range as intermediate spectra). Motivated by criticality safety needs, this work performs various benchmarks for uranium of various enrichment levels as well as plutonium.

The MCNP's precision could be limited by the cross section data in Evaluated Nuclear Data Files (ENDF), which it uses as input. To understand the scale of these possible discrepancies one can compare the nuclear cross section output from ENDF to the experimental data that they are based on. Figure 6 shows plots of ENDF cross sections along with experimental data for the four isotopes of plutonium, showing an overall good agreement.

To further strengthen the confidence in the ENDF, the experiments described in Losko et al.[4]

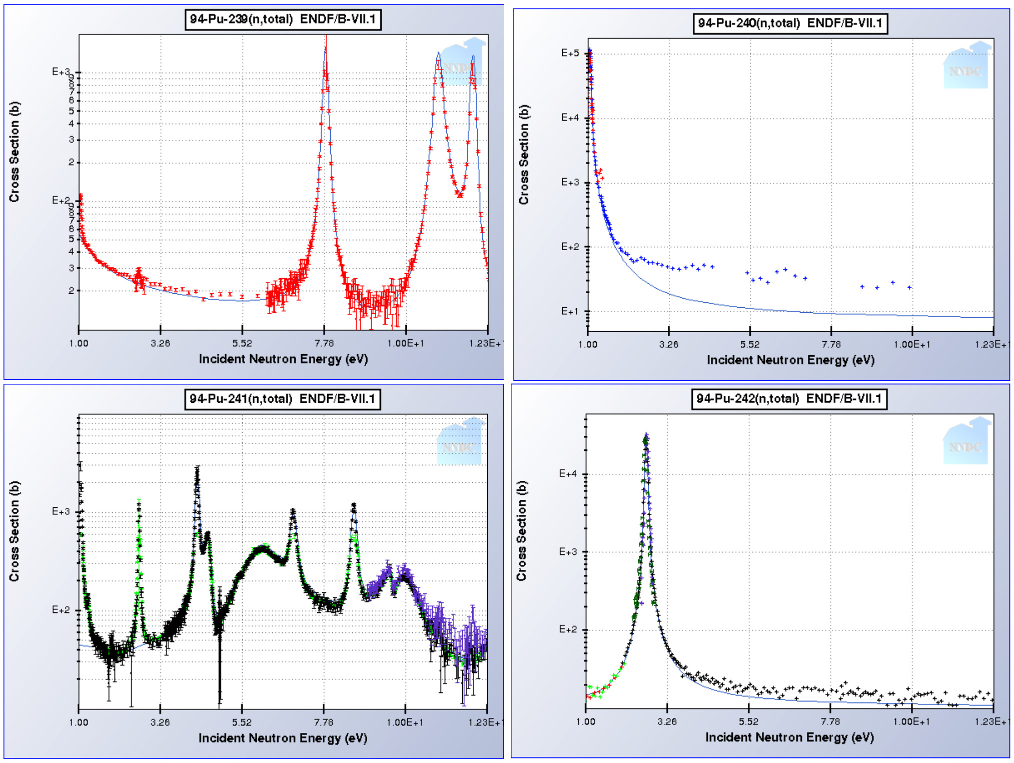

Supplementary Figure 6: Comparison of ENDF/B-VII.1 cross sections for (n,total) reaction to experimental data. The experimental data are plotted as points, and the error bars refer to one standard deviation of uncertainty. The comparison shows an overall good agreement. Some under-estimation (by a factor of $2\times$) is possible for ^{240}Pu. Plots and data taken from the National Nuclear Data Center (NNDC) Sigma web utility[16].

show a direct comparison of transmitted epithermal neutron spectral data in the 1-50 keV range to a theoretical fit. Figure 3 in that publication plots the experimental transmission data along with a transmission calculation, showing a strong agreement.

Supplementary References

1. Boivin, R., Koltonyuk, M., Munson, C. & Mayo, R. Time-of-flight neutral particle analyzer for alcator c-mod. *Review of scientific instruments* **68**, 982–985 (1997).

2. Harling, O. *et al.* The fission converter-based epithermal neutron irradiation facility at the massachusetts institute of technology reactor. *Nuclear science and engineering* **140**, 223–240 (2002).

3. Lisowski, P., Bowman, C., Russell, G. & Wender, S. The los alamos national laboratory spallation neutron sources. *Nuclear Science and Engineering* **106**, 208–218 (1990).

4. Losko, A. *et al.* Energy-resolved neutron imaging for interrogation of nuclear materials. In *Advances in Nuclear Nonproliferation Technology and Policy Conference* (2016).

5. Herrera, M. S., Moreno, G. A. & Kreiner, A. J. New method to evaluate the 7 li (p, n) 7 be reaction near threshold. *Nuclear Instruments and Methods in Physics Research Section B: Beam Interactions with Materials and Atoms* **349**, 64–71 (2015).

6. IBA Dynamitron. http://www.iba-industrial.com/accelerators#dynamitron-e-beam-accelerator. Accessed: 2018-02-21. Copyright date: 2014.

7. Matsuyama, S. *et al.* Upgrading of the 4.5 mv dynamitron accelerator at tohoku university for microbeam and nanobeam applications. *Nuclear Instruments and Methods in Physics Research Section B: Beam Interactions with Materials and Atoms* **267**, 2060–2064 (2009).

8. Mark, J. C., von Hippel, F. N. & Lyman, E. Explosive properties of reactor-grade plutonium. *Science & Global Security* **17**, 170–185 (2009).

9. McConn, R. J., Gesh, C. J., Pagh, R. T., Rucker, R. A. & Williams III, R. Compendium of material composition data for radiation transport modeling. Tech. Rep., Pacific Northwest National Laboratory (PNNL), Richland, WA (US) (2011).

10. Mamontov, E. Wide-angle mechanical velocity selection for scattered neutrons in inelastic neutron spectrometers. *Nuclear Instruments and Methods in Physics Research Section A: Accelerators, Spectrometers, Detectors and Associated Equipment* **759**, 83 – 91 (2014).

11. Briesmeister, J. F. *et al.* Mcnptm-a general monte carlo n-particle transport code. *Version 4C, LA-13709-M, Los Alamos National Laboratory* 2 (2000).

12. Tremsin, A. S., Feller, W. B. & Downing, R. G. Efficiency optimization of microchannel plate (mcp) neutron imaging detectors. I. Square channels with 10B doping. *Nuclear Instruments and Methods in Physics Research Section A: Accelerators, Spectrometers, Detectors and Associated Equipment* **539**, 278 – 311 (2005).

13. Yamazaki, A. *et al.* Neutrongamma discrimination based on pulse shape discrimination in a ce:licaalf6 scintillator. *Nuclear Instruments and Methods in Physics Research Section A: Accelerators, Spectrometers, Detectors and Associated Equipment* **652**, 435 – 438 (2011).

14. Totsuka, D. *et al.* Performance test of si pin photodiode line scanner for thermal neutron detection. *Nuclear Instruments and Methods in Physics Research Section A: Accelerators, Spectrometers, Detectors and Associated Equipment* **659**, 399–402 (2011).

15. Mosteller, R. D., Brown, F. B. & Kiedrowski, B. C. An expanded criticality validation suite for mcnp. *Transactions of the American Nuclear Society* **104**, 453 (2011).

16. Evaluated Nuclear Data File Retrieval and Plotting. http://www.nndc.bnl.gov/sigma/. Accessed: 2018-02-23.
